# Any modality of renal replacement therapy can be a treatment option for Joubert syndrome

**DOI:** 10.1038/s41598-020-80712-4

**Published:** 2021-01-11

**Authors:** Yoko Takagi, Kenichiro Miura, Tomoo Yabuuchi, Naoto Kaneko, Kiyonobu Ishizuka, Mariko Takei, Chikage Yajima, Yuka Ikeuchi, Yasuko Kobayashi, Takumi Takizawa, Masataka Hisano, Yoshinori Tsurusaki, Naomichi Matsumoto, Motoshi Hattori

**Affiliations:** 1 Department of Pediatric Nephrology, Tokyo Women’s Medical University, 8-1, Kawada-cho, Shinjuku-ku, Tokyo, 162-8666 Japan; 2 Department of Pediatrics, Gunma University Graduate School of Medicine, Maebashi, Gunma Japan; 3 Department of Nephrology, Chiba Children’s Hospital, Chiba, Japan; 4 Department of Human Genetics, Yokohama City University Graduate School of Medicine, Yokohama, Kanagawa Japan; 5 Faculty of Nutritional Science, Sagami Women’s University, Sagamihara, Kanagawa Japan

**Keywords:** Nephrology, Renal replacement therapy

## Abstract

Joubert syndrome (JS) is an inherited ciliopathy characterized by a distinctive cerebellar and brain stem malformation which is known as the “molar tooth sign” on axial brain images, hypotonia, and developmental delay. Approximately 25–30% of patients with JS have kidney disease and many of them progress to end-stage kidney disease (ESKD). However, there are few reports on the outcomes of renal replacement therapy (RRT) in patients with JS and ESKD. In this study, we clarified the clinical features, treatment, and outcomes of patients with JS who underwent RRT. We retrospectively analyzed the medical records and clinical characteristics of 11 patients with JS who underwent RRT between June 1994 and July 2019. Data are shown as the median (range). Gene analysis was performed in 8 of the 11 cases, and *CEP290* mutations were found in four patients, two had *TMEM67* mutations, one had a *RPGRIP1L* mutation, and one patient showed no mutation with the panel exome analysis. Complications in other organs included hydrocephalus in two cases, retinal degeneration in eight cases, coloboma in one case, liver diseases in four cases, and polydactyly in one case. Peritoneal dialysis (PD) was introduced in seven cases, with a median treatment duration of 5.4 (3.4–10.7) years. Hemodialysis was performed using arteriovenous fistula in two cases, and kidney transplantation was performed 9 times in eight cases. Only one of the grafts failed during the observation period of 25.6 (8.2–134.2) months. The glomerular filtration rate at the final observation was 78.1 (41.4–107.7) mL/min/1.73 m^2^. The median age at the final observation was 13.4 (5.6–25.1) years, and all patients were alive except one who died of hepatic failure while on PD. Any type of RRT modality can be a treatment option for patients with JS and ESKD.

## Introduction

Joubert syndrome (JS) is a predominantly autosomal recessive ciliopathy characterized by a distinctive cerebellar and brain stem defect which is known as the molar tooth sign (MTS), hypotonia, and developmental delay^1^. JS was first reported in 1969 as a disorder based on neurological features, such as temporary hyperpnea, eye movement abnormalities, cognitive dysfunction, and cerebellar vermis hypoplasia^2^. The incidence of JS is reported to be 1:80,000–1:100,000^1^. Primary cilia abnormalities are considered to be the main cause. In addition to the neurological manifestations, symptoms also appear in the eyes, liver, kidneys, and skeleton^3^. It has been reported that 25–30% of patients with JS have kidney diseases, such as polycystic kidney disease and nephronophthisis^4–6^, which progress to end-stage kidney disease (ESKD). To date, there are few reports regarding renal replacement therapy (RRT) for JS patients who progressed to ESKD. Here, we report the clinical features, treatment, and outcomes of 11 patients with JS who underwent RRT.

## Results

### Patients’ clinical characteristics

Table 1 shows the patients’ clinical characteristics. The male-to-female ratio was 8:3, and sibling cases (patient numbers 2a and 2b) were included. Of the 8 patients who underwent genetic analysis, gene mutations in *CEP290*, *TMEM67*, and *RPGRIP1L* were found in four patients, two patients, and one patient, respectively. We did not identify any mutation in the 172 genes analyzed with panel exome sequencing in the remaining one patient.

**Table 1:** Clinical characteristics of the patients.

Patient no	Sex	Gene	Mutation	Kidney US	Hypertension before ESKD	Specific type of kidney disease	Hydro-cephalus	Retinal degene-ration	Colo-boma	Liver disease	Poly-dactyly	IQ*	Develop-mental delay	Age at ESRD	Weight at ESKD	Hight at ESKD	First RRT	Out-come
1	male	*CEP290*	c.214G > T (p.E72*) c.6012-12 T > A	Bilateral enlarged kidney with hyperechogenicity	−	ARPKD-NPHP	−	+	−	−	−	NA	profound	1.8	7.1	74.6	PD	alive
2a	female	*CEP290*	c.5788A > T (p.K1930*) c.6012-12 T > A	Bilateral hyperechogenic kidney with loss of CMD	−	NPHP	−	+	−	−	−	NA	profound	7.4	19.6	116.8	PD	alive
2b	male	*CEP290*	c.5788A > T (p.K1930*) c.6012-12 T > A	Bilateral hyperechogenic kidney with loss of CMD	−	NPHP	−	+	−	−	−	NA	profound	8.2	24.8	120.3	PD	alive
3	male	*CEP290*	c.6271-8 T > G c.6012-12 T > A	Bilateral hyperechogenic kidney with loss of CMD	−	NPHP	−	+	−	−	−	NA	profound	6.1	13.3	93	PD	alive
4	male	*TMEM67*	c.329A > G (p.D110G) c.2322+5delG	Bilateral enlarged cystic kidney	−	ARPKD-NPHP	−	+	−	+	−	56	mild	9.2	18.3	112.3	PD	alive
5	male	*TMEM67*	c.329A > G (p.D110G) c.579_580delAG (p.G195Ifs*13)	Bilateral enlarged cystic kidney	+	ARPKD-NPHP	−	−	+	+	−	51	mild	11.3	20.0	120.5	PEKT	alive
6	female	*RPGRIP1L*	c.86-2A > G c.231-2A > G	Bilateral enlarged cystic kidney with hyperechogenicity	+	ARPKD-NPHP	+	+	−	+	−	NA	profound	2.2	8.0	71	PD	alive
7	female	no mutation		Left polycystic kidney Right ectopic kidney	−	other	−	−	−	−	+	46	moderate	6.8	14.5	94.5	PEKT	alive
8	male	not done		Bilateral hyperechogenic kidney with loss of CMD	−	NPHP	+	+	−	−	−	NA	profound	11.3	14.9	110.5	PEKT	alive
9	male	not done		Bilateral hyperechogenic kidney with loss of CMD	−	NPHP	−	+	−	−	−	50	mild	15.3	48.2	156.8	PEKT	alive
10	male	not done		Bilateral enlarged cystic kidney	−	ARPKD-NPHP	−	−	−	+	−	NA	profound	2.7	8.8	82	PD	dead

*Patients in which Wechsler Intelligence Scale for Children testing was not possible due to severe developmental delay (designated as NA) were considered to have profound developmental delay.

*RRT* renal replacement therapy, *CMD* corticomedullary differentiation, *ARPKD* autosomal recessive polycystic kidney disease, *NPHP* nephronophthisis, *PKD* polycystic kidney disease, *NA* not applicable, *PD* peritoneal dialysis, *PEKT* preemptive kidney transplantation; 2a and 2b are siblings.

**Figure 1:**
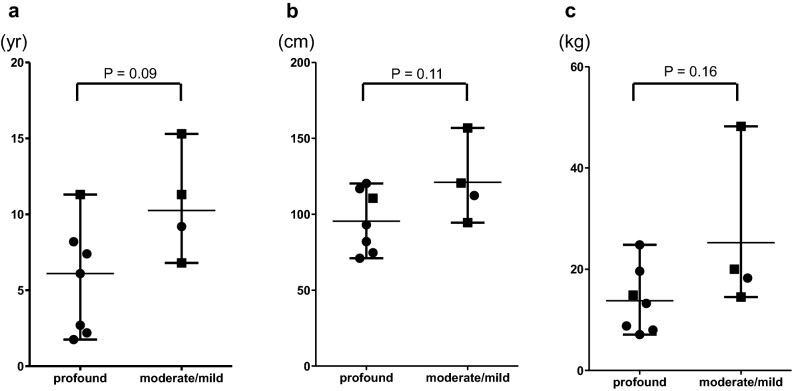
Age, height, and body weight at end-stage kidney disease in Joubert syndrome patients with profound and moderate/mild developmental delay. Age (**a**), height (**b**), and weight (**c**) at end-stage kidney disease are shown. Filled circles and filled squares denote patients who underwent peritoneal dialysis and preemptive living donor kidney transplantation as the initial RRT, respectively.

The specific type of kidney disease was classified as an overlap phenotype of autosomal recessive polycystic kidney disease and nephronophthisis (ARPKD-NPHP) in five patients and nephronophthisis (NPHP) in five patients. The remaining patient had a polycystic left kidney and an ectopic right kidney. Complications in other organs included hydrocephalus in two patients, retinal degeneration in eight, coloboma in one, liver disease in four, and polydactyly in one. The four patients with *CEP290* gene mutations showed retinal degeneration, and the two patients with *TMEM67* gene mutations had liver diseases. Developmental tests showed that seven patients had profound developmental delay, one had moderate delay, and three had mild delay. The four patients with *CEP290* gene mutations had profound developmental delay, while the two patients with *TMEM67* gene mutations had mild developmental delay. The median age at ESKD was 7.4 years (range 1.8–15.3) for all patients. The age at ESKD for the seven patients with profound developmental delay was 6.1 (1.8–11.3) years old, a younger age than the 10.3 (6.8–15.3) years for the patients with moderate/mild developmental delay, although there were no statistically significant differences between the two groups (Fig. 1a). In addition, the height and weight of the patients in the profound group tended to be lower compared with those of the patients in the moderate/mild group at the time of ESKD (Fig. 1b, c).

The treatment decision made for the first RRT was as follows (Table 1): three patients (patient number 5, 7, 9) of the four patients with moderate/mild developmental delay underwent preemptive living donor kidney transplantation (LDKT), while one (number 4) underwent peritoneal dialysis (PD). Six (numbers 1, 2a, 2b, 3, 6 and 10) of the seven patients with profound developmental delay underwent PD, and the remaining patient (number 8) underwent preemptive LDKT. As shown in Fig. 1, there was a tendency to select PD over preemptive kidney transplantation for young and small children and those with profound developmental delay. All patients were alive at the latest observation except for one who died of hepatic failure due to hepatic fibrosis while undergoing PD (Table 1).

### Outcomes of renal replacement therapy

**Figure 2:**
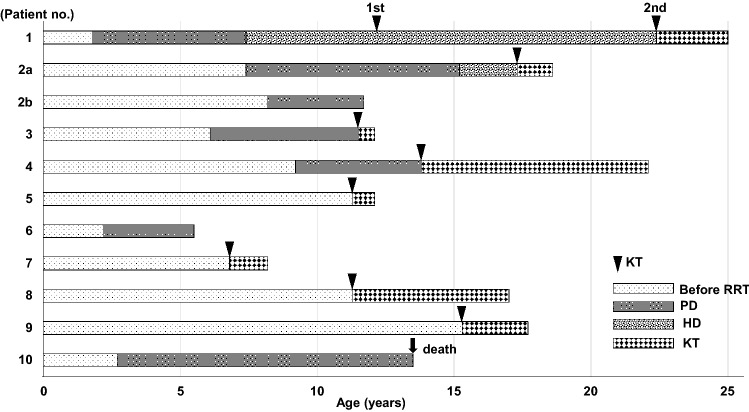
Clinical course of each patient. *KT* kidney transplantation, *RRT* renal replacement therapy, *PD* peritoneal dialysis, *HD* hemodialysis.

Figure 2 shows the clinical course of RRT in each case. The median age at the last observation was 13.4 years (range 5.6–25.1). PD was introduced as the first RRT for seven patients, and the median PD duration was 5.4 years (range 3.4–10.7). Two of the seven patients underwent deceased-donor kidney transplantation (DDKT), and two underwent DDKT after transitioning from PD to hemodialysis. The remaining three patients continued undergoing PD. The incidence of bacterial peritonitis was 0.07 cases/person-year in our patients. Three patients (numbers 3, 6 and 10) experienced recurrent catheter exit site infection. Hemodialysis was introduced as the second RRT for two due to ultrafiltration failure with the PD therapy. They underwent maintenance dialysis using an arteriovenous fistula in the outpatient clinic without significant complication during hemodialysis, although one of the patients required occasional sedation.

Three patients have not yet undergone kidney transplantation. Patient 2b is currently registered on the waiting list for DDKT. Preparations for LDKT are currently underway for patient 6. However, the patient’s indication for kidney transplantation still needs to be carefully evaluated because she has severe comorbidities and requires management with a ventriculoperitoneal shunt for her hydrocephalus. In the case of patient 10, kidney transplantation was not performed because of liver complications, and he eventually died of hepatic failure at the age of 13.4 years.

Table 2 shows the results of the nine kidney transplantations in eight patients. The median age at the time of transplantation was 12.6 (6.8–22.3) years. DDKT was performed in 5 operations with four patients (number 1, 2a, 3, and 4), and LDKT was performed in four patients (number 5, 7, 8, and 9). The immunosuppressive regimen consisted of basiliximab, methylprednisolone, a calcineurin inhibitor, and mycophenolate mofetil in all cases^13^. The median observation period after transplantation was 25.6 (8.2–134.2) months, and all grafts survived except for the first kidney transplant in patient 1 who underwent the initial DDKT at the age of 12 years, although he required transplant nephrectomy within 2 days because of transplant kidney artery thrombosis. The patient continued maintenance hemodialysis for the following 10 years until he underwent the second DDKT. Since then, the patient has been well managed. Patient 7 experienced acute T-cell mediated rejection at on day 48 postsurgery but improved with steroid pulse therapy. The patient also had a bacterial urinary tract infection and adenovirus nephritis. During the first year after transplantation, one patient experienced cytomegalovirus disease, and three patients presented cytomegalovirus infection requiring antiviral therapy. The overall incidence of infection was 0.75 cases/person-year. The median estimated glomerular filtration rate at the time of the final observation was 78.1 (range 41.4–107.7) mL/min/1.73 m^2^.

**Table 2:** Clinical characteristics of the transplanted patients.

Patient no	Age at transplant	Donor type of transplant	ABO compatibility	Immunosuppressive regimen	Period of observation after transplant (mo)	Rejection	Infection*	eGFR at the latest follow up (ml/min/1.73 m^2^)
1 (1st)	12.0	DD	Compatible	Tac, MMF, mPSL, basiliximab	2 days	No	None	Graft loss
1 (2nd)	22.3	DD	Compatible	Tac, MMF, mPSL, basiliximab	32.9	No	None	49.0
2a	17.2	DD	Compatible	Tac, MMF, mPSL, basiliximab	17.1	No	None	107.7
3	11.5	DD	Compatible	Tac, MMF, mPSL, basiliximab	8.2	No	CMV disease	61.7
4	13.8	DD	Compatible	Tac, MMF, mPSL, basiliximab	134.2	No	CMV infection	83.0
5	11.3	LD**	Compatible	Tac, MMF, mPSL, basiliximab	10.2	No	CMV infection	89.5
7	6.8	LD**	Compatible	CsA, MMF, mPSL, basiliximab	18.2	Yes	UTI CMV infection Adenovirus nephritis	98.8
8	11.3	LD**	Compatible	CsA, MMF, mPSL, basiliximab	76.2	No	None	73.2
9	15.3	LD**	Compatible	Tac, MMF, mPSL, basiliximab	43.5	No	None	41.4

*Infection during the first year after kidney transplantation.

**Patients 5, 7, 8, and 9 received preemptive kidney transplantation.

*DD* deceased donor, *LD* living donor, *Tac* tacrolimus, *MMF* mycophenolate mofetil, *mPSL* methyl prednisolone, *CsA* cyclosporin A, *CMV* cytomegalovirus, *UTI* urinary tract infection.

## Discussion

Few reports have been published on patients with JS and ESKD. In 2017, Fleming et al. retrospectively reviewed 97 patients with JS and reported 29 patients with JS and kidney disease^6^. Of the 13 patients with ESKD, 11 underwent kidney transplantation, but the details of the clinical course were not provided. To our knowledge, ours is the first report to describe the clinical features, treatment, and outcomes of patients with JS who underwent RRT.

Thirty-five genes have been identified as responsible for JS. Among them, *CEP290, NPHP1, AHI1, OFD1, RPGRIP1L, CC2D2A, TMEM67, TMEM216, TMEM138,* and *TMEM237* have been reported to be associated with kidney disease^1, 6, 14, 15^. Studies have also reported that the frequency of responsible genes varies by race, with *TMEM216* mutation occurring most often in Ashkenazi Jews^16^, *C5orf42* in the Dutch^17^, and *C5orf42, CC2D2A, NPHP1*, and *TMEM231* in French Canadians^18^. Suzuki et al. performed genetic analysis in 30 JS families and reported that mutations in *CEP290* and *TMEM67* were common among Japanese families^12^. The major phenotypes in our patients were consistent with those in previous reports indicating that the *CEP290* mutation is correlated with retinal degeneration and kidney disease, *TMEM67* is correlated with liver disease, and *RPGRIP1L* is correlated with kidney disease^1^. Furthermore, Fleming et al. reported that in 13 cases of ESKD, there were six cases with *TMEM67* mutations and four with *CEP290* mutations^6^. Taken together, these findings suggest that *CEP290* and *TMEM67* mutations are common in patients with JS and ESKD.

PD was more frequently selected as the initial RRT for the patients with profound developmental delay, whereas preemptive LDKT was often selected for the patients with moderate/mild developmental delay (Table 1). The age at ESKD tended to be younger in the patients with profound developmental delay than in those with moderate/mild developmental delay. The anthropometric measurements at the time of ESKD suggested that patients with profound developmental delay were smaller than those with moderate/mild developmental delay (Fig. 1). The selection of initial RRT can be affected by the age and anthropometric measurements at ESKD and the level of developmental delay.

In this study, PD was introduced in seven cases, while hemodialysis was introduced in two cases during the overall clinical course. PD and hemodialysis were performed without major complications in most of the patients. There have been several case reports regarding the introduction of dialysis to patients with JS and ESKD. Assadi reported on a patient with JS who was started on PD at 10 days of age, was changed to hemodialysis at 14 months due to refractory bacterial/fungal peritonitis, and who underwent DDKT at 2 years of age^19^. Sonmez et al. also reported on two patients with JS and ESKD, one of whom was administered hemodialysis at 5 years of age, while the other was administered PD at 22 months of age. Their RRT was continued without problem until the ages of nine and four years, respectively^20^. In our case series, the seven patients who underwent PD were generally well managed, although one patient developed peritonitis. The incidence of PD-related peritonitis has been reported in many countries, and the frequency varies depending on the PD population. Pirano et al. compiled several reports and reported that the incidence of PD-related peritonitis was 0.06–1.66 cases/person-year overall^21^. The International Society for Peritoneal Dialysis guidelines state that the overall incidence of PD-related peritonitis is 0.18–0.2 cases/person-year^22^. In our study, the incidence of PD-related peritonitis in the patients with JS was 0.07 cases/person-year, which is comparable to that of other populations. PD was therefore considered an acceptable therapeutic option for patients with JS and ESKD. Two cases who underwent hemodialysis were well managed, while one case required occasional sedation. Hemodialysis might be a possible RRT for selected patients with JS and ESKD.

To date, there have been no detailed reports on kidney transplantation in patients with JS^6^. In our study, eight patients underwent kidney transplantation. Only one of the grafts failed, and graft function was well preserved during the median observation period of 25.6 months. One patient experienced transplant kidney artery thrombosis immediately after DDKT, leading to graft loss; however, the course of the secondary transplant has been uneventful. One patient experienced a rejection episode that improved with treatment. The incidence of infection was 0.75 cases/person-year during the first year of transplantation in our study. The incidence of infectious diseases during the first year of transplantation in 890 pediatric patients who underwent kidney transplantation from 2002 to 2015 in Japan was 0.79 cases/person year, which was equivalent to our results^23^. This finding indicates that kidney transplantation could be a feasible treatment option for patient with JS and ESKD. However, we need to carefully evaluate the systemic complications, such as hydrocephalus requiring a ventriculoperitoneal shunt or hepatic failure, when considering the indication for kidney transplantation.

Our study’s limitations include the fact that it was a retrospective study and that it involved a small number of cases due to the low incidence of the disease. The analysis was conducted at a tertiary care facility where most cases were referred for RRT, and patients with JS and ESKD who did not undergo RRT were not included in this study. Analyzing the indication for RRT is therefore beyond the scope of our study. Further studies with more cases are needed to confirm the validity and safety of each RRT modality and evaluate the genotype–phenotype correlation in patients with JS and ESKD. In conclusion, for patients with JS and ESKD, any type of RRT modality can be a treatment option.

## Methods

From June 1994 to July 2019, we clinically diagnosed patients with JS at the Department of Pediatric Nephrology in the Tokyo Women’s Medical University and the Department of Pediatrics in Gunma University, Japan. This study included 11 patients with JS who underwent RRT. Study enrollment required a clinical diagnosis of JS based on the pathognomonic MTS in the brain magnetic resonance imaging (MRI)^6^. Patient information included sex, genetic mutation, kidney ultrasound findings, hypertension before ESKD, type of kidney disease, presence/absence of hydrocephalus/retinal degeneration/coloboma/liver disease/polydactyly, level of developmental delay, intelligence quotient, age at ESKD, height/weight at ESKD, type of initial RRT, and RRT outcomes. Specific types of kidney disease were classified into NPHP, ARPKD-NPHP, and others. NPHP was defined as normal-or small-sized kidney with hyperechogenicity, loss of corticomedullary differentiation, and/or cystic lesions at corticomedullary junction on a kidney ultrasound^6^. ARPKD-NPHP is considered to present with refractory hypertension or kidney swelling before kidney dysfunction^6^. Liver disease was defined by elevated liver enzyme levels and/or increased echogenicity of the liver and/or splenomegaly on abdominal ultrasounds and/or the findings of congenital hepatic fibrosis confirmed by liver biopsy^6^. Retinal degeneration was based on typical findings during a retinal examination after pupil dilation^6^. Developmental delay was assessed using the Wechsler Intelligence Scale for Children (WISC) where 50–70 is considered mild, 35–49 is considered moderate, 20–34 is considered severe, and < 20 or cases in which WISC testing is not possible due to severe developmental delay is considered profound^7^. Post-transplant infections were defined as those that required hospitalization. Cytomegalovirus infection was defined as the virus isolation or the detection of viral proteins (antigens) or nucleic acid in any body fluid or tissue specimen regardless of symptom. Cytomegalovirus disease was defined as cytomegalovirus infection with attributable symptoms, such as fever, malaise, leukopenia, and/or thrombocytopenia, or that exhibited organ or tissue damage^8^. We calculated the estimated glomerular filtration rate using the revised Schwartz formula for patients aged 17 years or younger^9^ or the mean of values from the revised Schwartz formula and the Chronic Kidney Disease Epidemiology Collaboration (CKD-EPI) formula for patients aged 18–26 years^10, 11^. For the statistical analysis, we employed the Mann–Whitney U test.

No organs were procured from prisoners and all transplantations were performed at Tokyo Women’s Medical University and Gunma University, Japan.

Eight of the 11 cases underwent gene analysis, six of which underwent whole-exome sequencing (WES) at Yokohama City University. One case underwent WES at Kanagawa Children’s Medical Center. The remaining case underwent panel exome sequencing of 172 genes associated with congenital abnormalities of the kidney and urinary tract and nephronophthisis-related ciliopathies at Kobe University. For WES, a Genome Analyzer IIx sequencer (Illumina, San Diego, CA), Hiseq 2000, or 2500 platform (Illumina) was employed as previously described^12^.

### Ethical approval

This study was approved by the Ethics Committee of Tokyo Women’s Medical University and Gunma University (approval number: 4654-R, HS2019-237). Gene analysis was performed with the consent of the individuals or guardians. All research was performed in accordance with relevant guidance and regulations. Comprehensive written informed consent was obtained from all individual participants and/or guardians included this study.

## Data Availability

The datasets used and/or analysed during the current study are available from the corresponding author on reasonable request.

## References

[1] Melissa, P. & Ian, G. Joubert syndrome—GeneReviews®—NCBI Bookshelf. https://www.ncbi.nlm.nih.gov/sites/books/NBK1325/.

[2] Joubert M, Eisenring JJ, Robb JP, Andermann F (1999). Familial agenesis of the cerebellar vermis: a syndrome of episodic hyperpnea, abnormal eye movements, ataxia, and retardation. J. Child Neurol..

[3] Romani M, Micalizzi A, Valente EM (2013). Joubert syndrome: congenital cerebellar ataxia with the molar tooth. Lancet Neurol..

[4] Saraiva JM, Baraitser M (1992). Joubert syndrome: a review. Am. J. Med. Genet..

[5] Bachmann-Gagescu R (2015). Joubert syndrome: a model for untangling recessive disorders with extreme genetic heterogeneity. J. Med. Genet..

[6] Fleming LR (2017). Prospective evaluation of kidney disease in Joubert syndrome. Clin. J. Am. Soc. Nephrol..

[7] ICD-10 Version:2016. https://icd.who.int/browse10/2016/en.

[8] Kotton CN (2018). The third international consensus guidelines on the management of cytomegalovirus in solid-organ transplantation. Transplantation.

[9] Schwartz GJ (2009). New equations to estimate GFR in children with CKD. J. Am. Soc. Nephrol..

[10] Levey AS (2009). A new equation to estimate glomerular filtration rate. Ann. Intern. Med..

[11] Ng DK (2018). Combination of pediatric and adult formulas yield valid glomerular filtration rate estimates in young adults with a history of pediatric chronic kidney disease. Kidney Int..

[12] Suzuki T (2016). Molecular genetic analysis of 30families with Joubert syndrome. Clin. Genet..

[13] Miura K (2020). Individualized concept for the treatment of autosomal recessive polycystic kidney disease with end-stage renal disease. Pediatr. Transplant..

[14] Sayer JA (2006). The centrosomal protein nephrocystin-6 is mutated in Joubert syndrome and activates transcription factor ATF4. Nat. Genet..

[15] Utsch B (2006). Identification of the first AHI1 gene mutations in nephronophthisis-associated Joubert syndrome. Pediatr. Nephrol..

[16] Edvardson S (2010). Joubert syndrome 2 (JBTS2) in Ashkenazi Jews is associated with a TMEM216 mutation. Am. J. Hum. Genet..

[17] Kroes HY (2016). Joubert syndrome: genotyping a Northern European patient cohort. Eur. J. Hum. Genet..

[18] Srour M (2015). Joubert syndrome in French Canadians and identification of mutations in CEP104. Am. J. Hum. Genet..

[19] Assadi F (2007). Lack of NPHP2 mutations in a newborn infant with Joubert syndrome-related disorder presenting as end-stage renal disease. Pediatr. Nephrol..

[20] Sönmez F (2014). Development of end-stage renal disease at a young age in two cases with Joubert syndrome. Turk. J. Pediatr..

[21] Piraino B (2011). ISPD position statement on reducing the risks of peritoneal dialysis-related infections. Perit. Dial. Int..

[22] Li PKISPD, Recommendations P (2016). Update on prevention and treatment. Perit. Dial. Int..

[23] Hattori M (2018). Outcomes of pediatric ABO-incompatible living kidney transplantations from 2002 to 2015: an analysis of the Japanese kidney transplant registry. Transplantation.

